# Translations

**Published:** 1880-02

**Authors:** 


TRANSLATIONS.—We are able to begin the publication of translations from foreign journals in this issue, and are promised such improvements and discoveries as may occur or be made in the various branches of the profession on the Continent of Europe, as far as space will permit, in future issues of the PRACTITIONER. The translations in this number are made from recent European publications, by a most accomplished physician of this city, who will continue to give his valuable services from time to time, to this department of our enterprise. Others have also assured us of their aid and co-operation in this field, which will enable us to present the *crème de la crème* of the medical journals of France and Germany to our readers hereafter.

